# Pruritic Cutaneous Nematodiasis Caused by Avian Eyeworm *Oxyspirura* Larvae, Vietnam

**DOI:** 10.3201/eid2604.191592

**Published:** 2020-04

**Authors:** Do T. Dung, Nguyen T. Hop, Tran H. Tho, Yukifumi Nawa, Pham N. Doanh

**Affiliations:** National Institute of Malariology, Parasitology, and Entomology, Hanoi, Vietnam (D.T. Dung, N.T. Hop, T.H. Tho);; Khon Kaen University, Khon Kaen, Thailand (Y. Nawa);; Institute of Ecology and Biological Resources, Graduate University of Science and Technology, Vietnam Academy of Science and Technology, Hanoi (P.N. Doanh)

**Keywords:** pruritic lesions, cutaneous nematode infection, Oxyspirura, larvae, 18S ribosomal DNA, skin infections, zoonoses, parasites, Vietnam, avian eyeworm, nematodiasis

## Abstract

A 41-year-old man from Son La Province, Vietnam, sought care for disseminated prurigo-like skin lesions from which nematode larvae were emerging. We morphologically and molecularly identified the larvae as *Oxyspirura* sp. Our findings confirm this nematode species as a zoonotic pathogen for emerging disease.

Various nematode parasites are known to cause cutaneous lesions in humans. Some species, such as nonlymphatic filaria *Onchocerca vorvuls* and *Loa loa*, exploit skin tissues to become mature adults and reproduce (*1*,*2*). Other species, usually animal hookworms such as *Ancylostoma braziliense* and *A. caninum*, as well as other less common species (*2*,*3*), remain larval or immature and accidentally migrate into cutaneous tissues (*1*). Clinical manifestations of cutaneous parasitic infections include migratory nodular lesions or erythema when parasites are in the deeper part of subcutis and serpiginous creeping eruptions when worms migrate through or just under the epidermis. Regardless of clinical manifestation, skin lesions caused by nematode parasites tend to be focal, except when larva currens of *Strongyloides stercoralis* (*2*,*4*) are involved and cause disseminated strongyloidiasis. We describe a case of disseminated cutaneous nematodiasis caused by *Oxyspirura* larvae, adult nematodes of which are known as avian eyeworms.

## The Study

In July 2019, a 41-year-old man from Son La Province, northern Vietnam, came to the clinic of the National Institute of Malariology, Parasitology, and Entomology (Hanoi) with symptoms of disseminated pruritic erythema. The patient reported being of Thai ethnicity and told clinicians that he had pruritic lesions for several years. The patient used an herbal lotion from a local traditional medicine practitioner to treat the lesions for a year, but his symptoms did not resolve.

**Figure 1:**
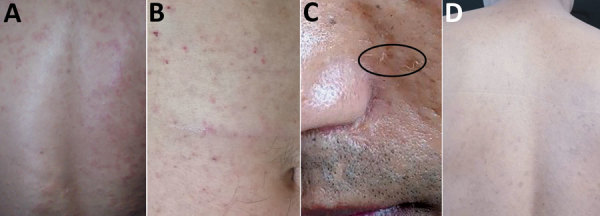
Lesions on the skin of a patient infected with *Oxyspirura* larvae, Vietnam. A, B) Lesions on the back and abdomen. C) Lesions on the face, with visible larvae (oval). D) Lesions on the patient’s back 2 months after treatment.

Physical examination revealed numerous erythematous, edematous, and pruritic skin lesions over his entire body skin, except for the soles of his feet. His back (Figure 1, panel A) and abdomen (Figure 1, panel B) were particularly affected. While the skin lesions were being examined, active larvae spontaneously migrated out (Figure 1, panel C) and even jumped out (Video 1) from the patient’s skin. 

**Video 1:** *Oxyspirura* larvae emerging from skin of a case-patient with severe pruritic skin lesions, Vietnam.

Hematology and biochemistry test results showed a slightly elevated total IgE of 171.2 IU/mL (reference range <100 IU/mL), but differential blood count, alanine transaminase, aspartate aminotransferase, creatinine, and other laboratory values were within reference limits. Ultrasonography and chest radiographs did not detect any abnormalities in lungs, liver, gallbladder, pancreas, kidneys, or bladder. A fecal sample was negative for nematode eggs and larvae. The patient noted that >3 of his neighbors had similar lesions, and some others suffered from sinusitis and had larvae emerge from their facial skin. The patient provided a video of the severe skin lesions of his neighbor (Video 2).

**Video 2:** *Oxyspirura* larvae emerging from skin of the neighbor of a case-patient with severe pruritic skin lesions, Vietnam. Video provided by the case-patient.

**Figure 2:**
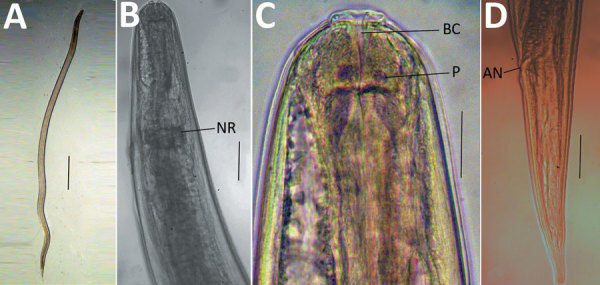
Microscopic images of *Oxyspirura* larvae collected from an infected patient, Vietnam. A) Whole body of *Oxyspirura* larvae; B, C) larvae anterior; D) larvae posterior. Scale bars indicate 100 μm in panel A, 50 μm in panels B–D. AN, anus; BC, buccal cavity; NR, nerve ring; P, papilla.

We collected 6 specimens of larvae that emerged from the patient’s skin for morphologic and molecular identification. The larvae were 800–850 µm long and 170–200 µm wide (Figure 2, panel A). The larvae had a nerve ring 212–250 µm from the anterior end (Figure 2, panel B), a clear buccal cavity (Figure 2, panel C), and an anus 300–350 µm from the posterior end (Figure 2, panel D). The larvae characteristics were similar to those of *Oxyspirura* spp. (*5*,*6*). 

We used 3 larvae for molecular identification by analyzing a partial 18S rDNA sequence. We extracted total DNA from the larvae by using a QIAamp DNA Stool Mini Kit (QIAGEN, https://www.qiagen.com). We successfully amplified an ≈900-bp region by PCR with primer pairs SSU18A and SSU26R (*7*). We directly sequenced both strands by using an Ab3730 (ThermoFisher, https://www.thermofisher.com) and obtained 3 identical 884-bp sequences. We deposited sequences into DDBJ (http://getentry.ddbj.nig.ac.jp; accession no. LC508119) and GenBank (accession no. LC508119). 

Consistent with the morphologic identification, a BLAST (http://blast.ncbi.nlm.nih.gov) search revealed that sequences from the larvae had a high similarity (96.6%) with that of *Oxyspirura petrowi* (accession no. LC316613). We downloaded 33 representative sequences with >93% similarity among the 100 highest similar sequences from GenBank and used these sequences to reconstruct a phylogenetic tree by using MEGA7 (https://www.megasoftware.net) and the maximum-likelihood method (*8*). The genomic sequence of the larvae from the patient clustered with *O. petrowi* at a high bootstrap value (93%), confirming that the larvae are of *Oxyspirura* species (Appendix Figure).

Eighty-four species are listed in the genus *Oxyspirura* (*9*). Most are avian eyeworms, and only 2 species were isolated from primates: *O. conjunctivalis* from a lemur and *O. youngi* from Patas monkeys (*6*). Our results confirm *Oxyspirura* larvae as a zoonotic pathogen and a cause of human disease. Despite the large number of nominal species of the genus, *O. petrowi* is the only species for which 18S rDNA sequences are available in GenBank, making identification of the larvae to the species level difficult. An investigation for adult nematode parasites in poultry raised in the patient’s community could help identify the zoonotic pathogen in this case. 

We treated the patient with albendazole (400 mg/d) for 14 days. His pruritis and lesions persisted but greatly improved after 2 months (Figure 1, panel D).

Skin lesions caused by nematode larvae vary depending on the causative pathogens. Cutaneous larva migrans caused by *A. caninum* canine hookworms or *A. braziliense* feline hookworms typically appear as multiple linear or serpiginous lesions on the feet, lower legs, and buttocks (*3*). *Gnathostoma* spp. larvae can cause either creeping eruption or migratory panniculitis (*2*,*10*). In Japan, Spirurina type X larvae in scintillant squids, now identified as the larvae of *Crassicauda giliakiana*, cause cutaneous creeping eruptions (*11*). A zoonotic canine filaria, *Dirofilaria repens*, causes nodular lesions on the skin of humans from Europe to South Asia (*12*). Although rare, larvae of the free-living nematode *Pelodera strongyloides* can infect humans and cause multiple pruritic skin lesions (*13*). Regardless of various clinical features, nematode larvae in humans usually cause focal skin lesions in limited areas (*2*). The case we report is an unusual example of disseminated pruritic lesions caused by *Oxyspirura* larvae.

For transmission, *Oxyspirura* species require arthropod intermediate hosts, such as cockroaches, crickets, and grasshoppers, to develop into infective third-stage larvae (*5*,*14*). The patient in our case affirmed he eats grasshoppers and crickets, which are potential intermediate hosts of the nematode and could have been the route of transmission. Eating insects is a common traditional custom in many countries (*15*), and cutaneous nematodiasis due to *Oxyspirura* larvae is likely in other locations. 

## Conclusions

We describe a case of systemic cutaneous larval nematodiasis caused by *Oxyspirura* sp. larvae in Son La Province, Vietnam. Because most members of this genus are parasites of birds, investigation for nematodes of poultry in this area and surrounding areas is needed to collect adult worms for species identification. Neighbors of the patient also had the same condition, which suggests that *Oxyspirura* sp. larvae could be a public health concern. Further investigations to determine potential intermediate hosts of this nematode, additional cases of cutaneous larval nematodiasis in the community, and sources of infection will enable the control of infections in animals and humans. 

Appendix Phylogenic tree of *Oxyspirura* larvae collected from the skin of a patient, Vietnam.
