# Supplementary figures and images for: Pruritic Cutaneous Nematodiasis Caused by Avian Eyeworm Oxyspirura Larvae, Vietnam
Source: Emerg Infect Dis. 2020 Apr;26(4):786–8. doi: 10.3201/eid2604.191592 (PMC7101080; doi:10.3201/eid2604.191592)

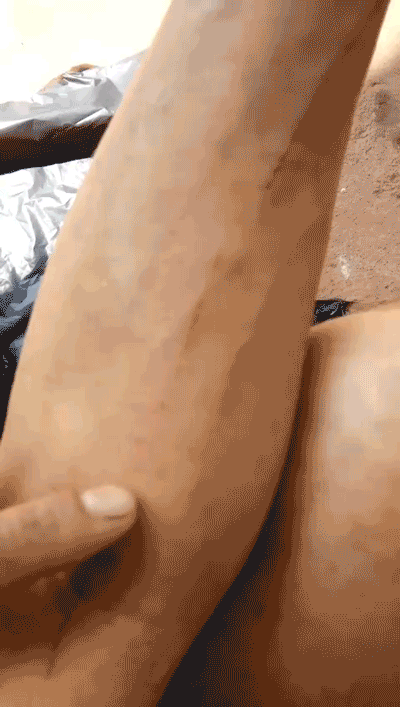

Supplement: Supplementary file 1 [file 19-1592-V1.gif]

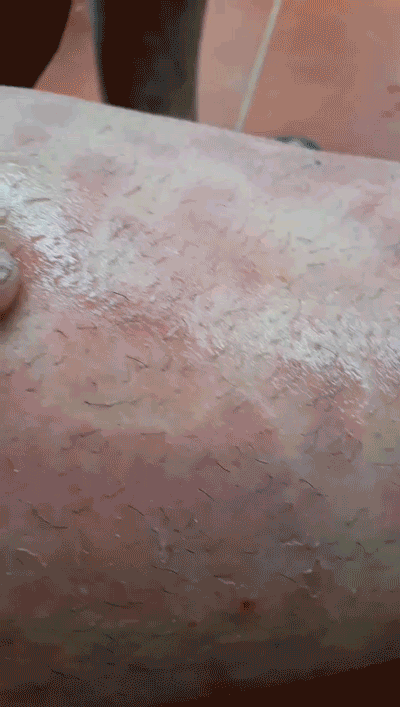

Supplement: Supplementary file 2 [file 19-1592-V2.gif]
