# Supplementary material for: Pruritic Cutaneous Nematodiasis Caused by Avian Eyeworm Oxyspirura Larvae, Vietnam
Source: Emerg Infect Dis. 2020 Apr;26(4):786–8. doi: 10.3201/eid2604.191592 (PMC7101080; doi:10.3201/eid2604.191592)
Supplement: Appendix — Phylogenic tree of Oxyspirura larvae collected from the skin of a patient, Vietnam. [file 19-1592-Techapp-s1.pdf]

Article DOI: https://doi.org/10.3201/eid2604.191592

Pruritic Cutaneous Nematodiasis Caused by Avian Eyeworm *Oxyspirura* Larvae, Vietnam

Appendix

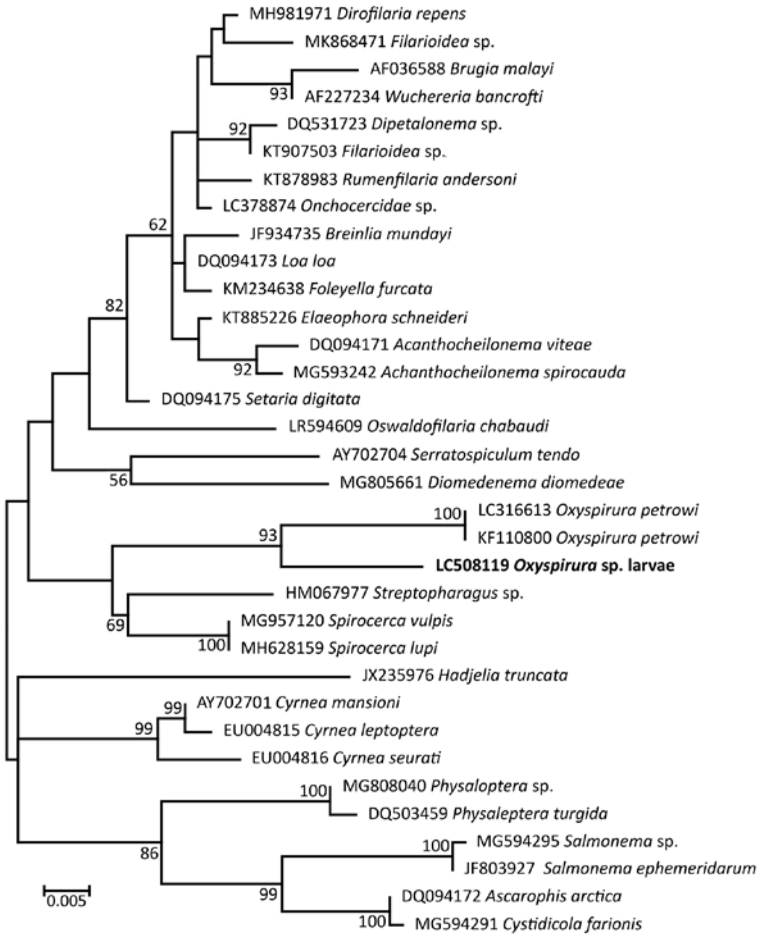

Appendix Figure. Molecular phylogenetic tree reconstructed from 18S rDNA sequences of *Oxyspirura* larvae from skin lesions of a patient, Vietnam. The evolutionary history was inferred by using the maximum likelihood method based on the Kimura 2-parameter model. A discrete Gamma distribution was used to model evolutionary rate differences among sites (+*G*). The rate variation model allowed some sites to be evolutionarily invariable (+*I*). All positions containing gaps and missing data were eliminated. Bootstrap values >50% are shown above branches. Bold text indicates nucleotide sequences obtained in this study. Scale bar indicates nucleotide substitutions per site.
